# Navigating Treatment Challenges: A Case Study on Refractory Psychosis in a Chronic MDMA (3,4-Methylenedioxymethamphetamine) User

**DOI:** 10.7759/cureus.59641

**Published:** 2024-05-04

**Authors:** Melissa N Litenski, Aoife B O'Reardon, Nicole Pabon, Melissa Hernandez, Yakov Niyazov, Jose Cruz

**Affiliations:** 1 Department of Psychiatry and Behavioral Health, Florida International University, Herbert Wertheim College of Medicine, Miami, USA; 2 Department of Psychiatry, Mount Sinai Medical Center, Miami, USA

**Keywords:** antipsychotics, mdma use, bipolar i disorder, alternative treatments, electroconvulsive therapy (ect), drug-induced catatonia, drug addiction, refractory psychosis

## Abstract

MDMA (3,4-methylenedioxy​methamphetamine), also known as Ecstasy, is a synthetic amphetamine with hallucinogenic and stimulant properties, which has become increasingly favored as a substance for recreational use. Despite its deceptive reputation as "safe," chronic MDMA use is associated with neuropsychiatric complications, including psychosis. We describe a case of a 23-year-old woman with chronic MDMA use disorder and childhood trauma, who presented with severe psychosis and catatonic features. While initial diagnostic possibilities included drug-induced psychosis and mood disorders, the patient's history and presentation supported a diagnosis of bipolar I disorder with psychotic features, which was exacerbated by MDMA use. Conventional antipsychotics failed to improve psychotic symptoms and led to worsening of catatonia, requiring electroconvulsive therapy (ECT) for improvement. Socioeconomic barriers hindered follow-up care, leading to an Emergency Department (ED) admission shortly after discharge. This case highlights the intricate interplay between substance use, psychiatric illness, and trauma, and showcases ECT's efficacy in severe psychosis. It emphasizes the necessity for comprehensive mental health services, especially for vulnerable populations, and calls for further research into MDMA's psychiatric effects and optimal treatment approaches for individuals with co-occurring substance use and psychiatric disorders.

## Introduction

MDMA (3,4-methylenedioxymethamphetamine), also known as Ecstasy, is a synthetic amphetamine that has both hallucinogenic and stimulant properties [1]. Presently, it has reached epidemic levels as a recreational street drug. According to the 2021 National Survey on Drug Use and Health, among people aged 12 years or older, 0.8% (or around 2.2 million people) reported using MDMA in the past 12 months [2]. Subjective effects of MDMA encompass heightened mood, enhanced self-assurance, and increased sensory receptivity. These effects are often accompanied by a serene sensation, enhanced insight, empathy, and a sense of connection to others, giving it a deceptive reputation as “safe” [3]. Yet, case reports as early as the 1990s have been documenting the neuropsychiatric complications following MDMA use [4-6]. Adverse psychiatric symptomatology associated with MDMA use includes panic attacks, depression, suicidal ideation, flashbacks, psychosis, and severe paranoia [7].

The long-term effects of chronic MDMA use extend beyond psychiatric symptoms, with emerging evidence suggesting potential neurotoxicity [8]. MDMA use has been associated with alterations in serotonin transporter density and function and serotonin depletion, leading to persistent changes in the serotonin system and increased striatal glutamate [9]. This surge in glutamate levels is consequential, as it has been correlated with an augmented release of dopamine [10]. This rise in dopamine levels is noteworthy, as it is linked to disruptions in dopamine regulation, a factor associated with the occurrence of psychosis [11].

This case is of a young woman with a significant history of MDMA use disorder on a substrate of childhood trauma and underlying history of bipolar disorder, who presented with a refractory psychosis and development of drug-induced catatonic features. Despite non-response to serial neuroleptic medications, the patient’s psychotic illness ultimately responded to a course of electroconvulsive therapy (ECT).

This case, which was presented at the University of Central Florida MedPACt’s Global Health Conference XIII: Addiction: A Global and National Health Crisis on January 13th, 2024, sheds light on the complexities of MDMA-induced psychosis and underscores the importance of considering novel treatment approaches, such as ECT, in cases of refractory psychosis.

## Case presentation

The patient is a 23-year-old adult female who initially presented with an unknown identity and was Baker Acted by the police after being found running along a roadway. Upon initial assessment in the ED, she appeared to be altered and possibly psychotic and therefore could not provide a coherent history. On clinical observation, she was noted to have incongruent sporadic laughter and to be responding to internal stimuli. Some of her initial features were suggestive of catatonia, including mutism with verbal communication limited to stating her name and negativism with a lack of response to any questions. Initial workup included an unremarkable serum and urine toxicology, sexually transmitted diseases panel, and brain computed tomography (CT); however, she was positive for COVID-19, and a complete metabolic panel showed hypokalemia (2.4 mmol/L). She was admitted to the medical floor for the management of COVID-19 symptoms and hypokalemia. During her medical admission, she was followed by the psychiatry team. On the 10th day, she was transferred to the behavioral health unit.

A collateral history was obtained on day 10, in which the patient’s mother revealed a history of psychiatric illness and substance abuse since her late teenage years. The information available indicated that her substance use history initially involved intermittent marijuana and cocaine use, then persistent MDMA, and, more recently, experimentation with a drug concoction called “tusi” (an admixture of MDMA, methamphetamine, cocaine, and opioids). In addition, two years prior to presentation, she experienced a lengthy 40-day hospitalization in her native country due to a manic episode, initially suspected to be linked to substance use, which led to a diagnosis of bipolar I disorder. The symptoms of this episode included religious preoccupation, lack of sleep, and excessive spending. Previous medications during this period included valproic acid, risperidone, and quetiapine. However, no further details regarding the dosing or length of time she was on these medications were available. It appears that the patient failed to follow up subsequently with outpatient treatment after that hospital stay.

The patient’s mother stated that there was a history of head trauma at age 10 but was unable to give any more information. Her mother did not report any psychiatric problems in childhood or any issues with development. Regarding sexual history, the patient stated that she identifies as heterosexual but had a few sexual encounters with women. Additionally, the patient endorsed sexual abuse by her maternal grandfather, which was confirmed by the family. The patient also indicated sexual abuse by her sister and her sister’s husband, which was denied by the family. Later, during her hospital stay, when she was able to communicate, the patient also reported recurrent emotional and physical abuse in childhood. It is important to mention that the patient had become estranged from her family for a period of time during adulthood. However, most recently, she had returned to live with her mother before this hospitalization, following her unemployment from her job as a waitress in a restaurant. According to the mother, an acquaintance informed her that the patient had lost her job due to drug use. In regard to a family history of psychiatric illness, the mother indicated that she believed the patient’s birth father committed suicide. However, this was unconfirmed because the birth father was not active in the patient's life.

Throughout the patient’s prolonged hospitalization, she exhibited a distinctive array of behaviors, which diverged from typical patterns associated with drug-induced psychosis. These behaviors included episodes of agitation, such as pounding on windows, forcefully slapping doors, and attempting to elope from the psychiatric floor. She also exhibited many features typical of bipolar disorder, including impulsivity, disinhibition, and sexual preoccupations. Also, mood instability was evident in her alternating states of laughter, withdrawal, and inconsolable crying. She had episodes of physiological dyscontrol in the form of enuresis and emesis. Additionally, nightly observations documented instances of self-conversation and pacing around the unit while unclothed. Of particular interest was the patient’s mutism when directly addressed; this was interspersed with brief moments of lucidity during which she shared distorted perceptions of her surroundings. These features collectively align with the definition of delirium, characterized by disturbances in attention and awareness, fluctuating cognitive deficits, and altered perception of reality.

Differential diagnosis

The patient initially presented to the unit displaying clear signs of psychosis, as detailed above. According to parental collateral history, the patient had previously been diagnosed with a manic episode in the context of chronic MDMA use disorder. The onset of psychosis on this occasion was also believed to be correlated with chronic drug use, with MDMA now combined with other illicit drugs (ketamine, methamphetamine) in a novel form, called “tusi.” Her overall worsening course of illness was likely in the context of non-adherence to treatment.

Initial diagnostic possibilities comprised bipolar I with psychotic features, drug-induced psychosis, schizophrenia, and schizoaffective disorder. In her earlier background, collateral history also revealed a history of extensive childhood abuse, suggesting a likely comorbid diagnosis of post-traumatic stress disorder (PTSD). Additionally, considerations were made regarding the possibility of a comorbid personality disorder, such as borderline personality disorder. This was particularly considered in light of the patient’s suicidality, impulsivity, and frequent displays of temper while hospitalized and information provided by collaterals.

Given the patient's well-groomed appearance and maintained state upon admission, coupled with collateral information indicating her functional status in society, including prior employment and social connections, as well as a lack of negative symptoms, the likelihood of schizophrenia or schizoaffective disorder as diagnosis decreased.

Provisionally, given the patient’s prior reported episode of mania coupled with psychotic symptoms, the absence of isolated psychosis without accompanying manic episodes, and the current presentation of hallucinations, paranoia, and disorganized thinking, the most probable clinical diagnosis was determined to be bipolar I disorder with psychotic features. This diagnosis was likely precipitated and exacerbated by concurrent MDMA substance use disorder, PTSD, and a personality disorder.

Treatment

In all, this patient spent eight weeks in the inpatient psychiatric unit following the three weeks on the medical floor. During this time, she required intensive medication management and underwent multiple medication trials. Treatment was started on the medical floor with haloperidol 5 mg Q6 PRN and lorazepam 2 mg Q6 PRN. As those were insufficient in addressing her agitation, she was additionally started on lorazepam 1 mg TID IV. For psychosis management, initial neuroleptic treatment began with risperidone at 0.5 mg BID. Risperidone was subsequently titrated up to 1.5 mg over the next two weeks. However, after two weeks, risperidone had to be discontinued because of uptrending liver function tests (LFTs). The medical team was consulted and cleared the patient to continue with further recommended psychiatric management, which included starting on olanzapine at 5 mg BID. To avoid potential respiratory depression, lorazepam was transitioned to 0.5 mg TID tablet form. Additionally, given the history of a prior manic episode, valproic acid at 500 mg TID was also added to her regime.

It is notable that during the second and third weeks of hospitalization, there were worsening catatonic-type features such as echolalia, echopraxia, disorientation, stereotypical movements, and prolonged staring. Initially, neuroleptic medication was briefly withheld, while neuroleptic malignant syndrome was ruled out. While labs showed a modest rise in creatine phosphokinase (CPK) of 197, a partial response to a lorazepam 2 mg challenge was supportive of a diagnosis of drug-induced catatonia. Therefore, this transient rise in CPK appears to have simply been due to a parenteral administration of haloperidol. Following this incident, lorazepam was continued at 0.5 mg TID. However, since lorazepam was only partially effective on a transient basis, neuroleptic medication was then restarted in light of worsening of psychosis. In terms of PRN medications throughout hospitalization, this patient required haloperidol 5 mg injection Q6 to manage agitation.

Regarding further neuroleptic management over the course of the next two weeks, olanzapine was titrated up to 20 mg, and valproic acid was changed to once nightly administration at 1,500 mg QPM. However, despite titrating olanzapine to maximally tolerated therapeutic doses, the patient remained floridly psychotic. Consequently, due to the lack of improvement, olanzapine was discontinued. Subsequently, quetiapine was initiated at 300 mg QPM during week 5, while valproic acid continued unchanged. Over the next four weeks, quetiapine was gradually titrated up to 700 mg QPM in continued combination with valproic acid 1,500 mg QPM. Despite intensive medication management, she remained psychotic and experienced significant sedation and weight gain. The significant sedation necessitated a dose decrease of quetiapine from 700 mg QPM to 500 mg QPM during week 9.

**Figure 1:**
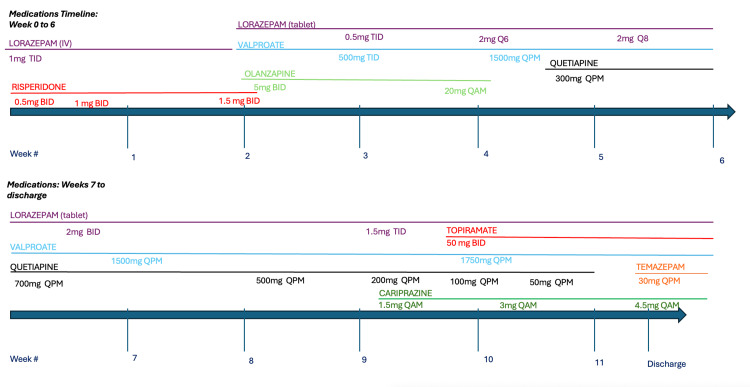
Timeline and composition of scheduled medication regimen during the inpatient stay

At week 8, given the ongoing lack of response to multiple serial neuroleptic trials, at maximum dosages and in various combinations, the decision was made to proceed to a trial of ECT given its established effectiveness in treating catatonic states. In all 12 sessions of ECT, bilateral stimulation was administered at a rate of three sessions per week. The patient responded well to ECT with a clearcut improvement in her psychotic state. In terms of medication management, quetiapine continued to be slowly titrated down to 50 mg QPM during the remainder of the hospital stay due to concerns regarding sedation. Cariprazine was added at week 10 for the augmentation of bipolar symptoms and titrated up to 4.5 mg QAM by discharge. Despite the initiation of cariprazine the patient continued to experience significant breakthrough manic symptoms. Therefore, during week 10, topiramate was added at 50 mg BID, and valproate was increased to 1,750 mg QPM. Treatment is further illustrated in Figure 1.

According to the patient's discharge notes, the Mental Status Examination revealed the following: the patient was cooperative with good eye contact and exhibited no abnormal movements. Speech was characterized by a slow yet well-articulated manner. The thought process appeared organized and followed a linear pattern. The patient denied experiencing hallucinations or suicidal ideations and demonstrated orientation to person, place, and time. Overall, the patient exhibited fair insight and judgment.

Follow-up

On day 81 of her psychiatric inpatient stay, she was discharged to the care of her grandmother. Extensive psychoeducation to the patient and her grandmother was given in regard to medication compliance, potential side effects, and the importance of life-long aftercare and follow-up. In the initial post-discharge planning, consideration was given to enrolling the patient in a partial hospitalization program. However, due to the patient's lack of health insurance and coming from a family of low socioeconomic status, it was determined that the patient could not afford continued treatment in such a manner. Instead, the patient was given follow-up instructions for a clinic located central to her discharge site, which offered sliding scale fees to assist with her continued care. Discharge medications were valproic acid 1750 mg daily, lorazepam 0.5 mg TID, topiramate 50 mg BID, temazepam 30 mg nightly for insomnia, and cariprazine 4.5 mg daily. Samples were provided of cariprazine, but the patient and her family were advised to go to the pharmacy to fill out the remaining prescriptions. Additionally, the patient was counseled on the benefits of psychotherapy and advised to pursue outpatient follow-up care, although no information is available regarding whether she did so.

Two days after discharge, the patient was brought to the ED by a guardian due to worsening psychotic symptoms secondary to medication non-compliance. The patient was Baker Acted by the ED physician. Per the Baker Act, “patient presented with odd/erratic behavior including tangential, laughing inappropriately, presenting with acute psychotic behavior. Patient unable to care for self, patient with disorganized thoughts and behaviors.” The patient’s toxicology panel was negative, and blood alcohol level was <10. The patient received 1 mg of benztropine mesylate and a 5 mg tablet of diazepam. The psychiatric evaluation concluded that the patient did not meet the criteria for involuntary inpatient psychiatric admission given that she did not present a danger to herself or others. Therefore, psychoeducation with the family and the patient was conducted, and she was discharged later that day. Additionally, the patient returned to the ED four days later for newly developed extrapyramidal symptoms. She was given diphenhydramine 50 mg IV and midazolam 2 mg IV. Symptoms improved, and the patient was discharged on the same day. A telephone follow-up was unsuccessful. In addition, records from the referred hospital indicate that the patient never followed up in their outpatient clinic.

## Discussion

This case highlights the complex interplay between substance use disorders, psychiatric illness, and traumatic experiences. The patient’s history of chronic MDMA use, compounded by childhood trauma and subsequent experimentation with a polydrug cocktail, led to a severe lengthy hospital stay and unconventional treatment-resistant psychotic episode. Despite intensive inpatient management, including multiple trials of antipsychotic medications, the patient's symptoms persisted and eventually evolved into drug-induced catatonia. The successful resolution of her psychotic symptoms with ECT highlights the importance of considering alternative treatments, particularly in cases of refractory psychosis. However, the challenges did not end upon discharge: despite thorough psychoeducation and a carefully planned outpatient follow-up, the patient's lack of health insurance and socioeconomic barriers hindered her ability to access continued care effectively.

Additionally, it is important to consider potential psychiatric toxicity associated with MDMA as it is both a common drug of abuse, and potentially in the near future, it may become available to clinicians as an FDA-approved treatment for PTSD (a new drug application for this approval is under review by the FDA currently). A recent case report by Virani et al. reports that typical clinical features of MDMA-induced psychosis included paranoid delusions, lack of verbal response, aggression, agitation, and suicidal ideation, as was the case here [6]. Another case report by Van Kampen and Katz also presented a case of MDMA-related psychosis with a resulting protracted psychotic course lasting three months despite cessation of MDMA use [12]. Similar overlaps in symptomatology were also seen in this case, including agitation, bizarre sexual behavior, and persistent delusional ideation regarding ideas of reference. In this case, we also observed that psychosis continued for an extended period despite cessation of drug use while in the hospital for several weeks.

Another novel feature of this case was the remarkable response to ECT in treating a severe psychotic illness that had previously failed to respond to multiple trials of neuroleptic medications. This highlights the potential efficacy of ECT as a viable treatment option when conventional approaches prove ineffective. Two particular clinical aspects of this case may have predisposed to the patient’s positive response to ECT. Firstly, the presence of catatonic features is a positive predictor of response to ECT, even in cases when lorazepam has been ineffective [13]. This suggests that ECT can effectively target and alleviate symptoms of catatonia, even in cases where other interventions have fallen short. Secondly, the underlying psychiatric diagnosis in this case, aside from the MDMA use disorder, appears to be bipolar I disorder, as evidenced by a prior manic episode. It is well established that ECT is an effective treatment for mood disorders with psychosis, whether they are unipolar or bipolar in nature [14]. Therefore, the bipolar aspect of the patient’s illness may have contributed to the favorable response to ECT. Moreover, while MDMA played a significant role in precipitating the psychotic disorder in this case, it is crucial to recognize that the patient’s psychiatric condition was not solely attributable to substance use. Rather, it was a complex interplay of underlying bipolar disorder and MDMA use disorder in which ECT emerged as a pivotal intervention, effectively addressing the psychotic symptoms stemming from both components of the patient’s illness.

This case highlights the need for comprehensive and accessible mental health services, particularly for individuals from disadvantaged backgrounds who are at higher risk of substance abuse and psychiatric disorders. Additionally, it highlights the danger of substance abuse for those individuals with underlying psychiatric illnesses. Furthermore, this case demonstrates the limitations of the current healthcare system in providing adequate support for such complex cases. Further efforts are needed to address these systemic issues and ensure that individuals, like the patient in this case, receive the comprehensive and ongoing care they need to manage their illness effectively and prevent further crises.

## Conclusions

In conclusion, this case illustrates the intricate interplay between substance use disorders, psychiatric pathology, and traumatic experiences. The patient's history of chronic MDMA use, compounded by childhood trauma and subsequent experimentation with a polydrug cocktail, led to a severe and lengthy hospital stay marked by a treatment-resistant psychotic episode. Despite intensive inpatient management, including multiple trials of antipsychotic medications, the patient's symptoms persisted. In addition, throughout these trials, she also developed drug-induced catatonia. The successful resolution of her psychotic symptoms with ECT stresses the importance of considering alternative treatments, particularly in cases of refractory psychosis. Furthermore, the challenges encountered post-discharge, including socioeconomic barriers, emphasize the urgent need for comprehensive and accessible mental health services, especially for vulnerable populations.

Additionally, this case prompts reflection on the potential psychiatric toxicity associated with MDMA, a commonly abused drug with emerging therapeutic potential. The patient's prolonged psychotic course despite cessation of drug use while hospitalized highlights the need for heightened awareness and understanding of MDMA-induced psychosis. Furthermore, the remarkable response to ECT in treating a severe and treatment-resistant psychotic illness underlines the potential efficacy of ECT as a viable treatment option when conventional approaches fail. Moving forward, it is imperative to address systemic issues within the healthcare system to ensure that individuals with complex psychiatric presentations receive the comprehensive and ongoing care they need to manage their illness effectively and prevent further crises.
